# Ecuadorian Journalists Mental Health Influence on Changing Job Desire: A Cross Sectional Study

**DOI:** 10.3390/ijerph181910139

**Published:** 2021-09-27

**Authors:** Byron Fernando Bustamante-Granda, Claudia Rodríguez-Hidalgo, María Aranzazu Cisneros-Vidal, Diana Rivera-Rogel, Claudia Torres-Montesinos

**Affiliations:** Department of Communication Sciences and Department of Psychology, Faculty of Social Sciences, Education and Humanities, Universidad Técnica Particular de Loja, Loja 110107, Ecuador; cvrodriguezx@utpl.edu.ec (C.R.-H.); macisneros@utpl.edu.ec (M.A.C.-V.); derivera@utpl.edu.ec (D.R.-R.); ctorres@utpl.edu.ec (C.T.-M.)

**Keywords:** journalists, emotional exhaustion, job change

## Abstract

Journalist’s mental health could predict their job change. This study aims at determining the prevalence of mental health issues and their association with perception of aptitude for covering emergencies and difficulty in seeing a corpse, and also to determine the mental health factors associated with job change. An ad hoc survey, GHQ-28 (Somatization, Anxiety-Insomnia, Social Dysfunction, Depression), MBI-P (Burnout, Emotional Exhaustion, Depersonalization, personal accomplishment) and Brief scale to diagnose Post-Traumatic Stress Disorder and Suicide Risk were applied to 196 journalists (female = 51.6%). Descriptive analysis, correlations (Pearson and Spearman), T-test and binary logistic regression were performed. It was found that one third part of journalists perceive themselves as having low aptitude to cover emergencies and difficulty in seeing a corpse, 17.3% would consider changing jobs and 42.1% could only access free mental health services. The most frequent mental health problems are: low personal accomplishment, emotional exhaustion and post-traumatic stress disorder (11.2 to 17.3%). People who want to change jobs present more: social dysfunction, depression, emotional exhaustion, depersonalization, low personal accomplishment, post-traumatic stress disorder and suicide risk. The two mental health factors associated with desire of changing jobs are high emotional exhaustion, and low personal accomplishment. These results guide the psychosocial risk prevention processes for journalists, as well as the training needs that universities could consider to protect the mental health of this vulnerable group.

## 1. Introduction

Crisis situations (i.e., natural disasters, social uprisings, armed conflicts, health crises) alter the daily life in our society; simultaneously, they are part of the media headlines, and communication professionals (henceforth journalists) are responsible for providing information to reduce uncertainty in society; or, on the contrary, to increase panic and chaos [1]. In addition to telling the facts, journalism also contributes by creating the frameworks of reference for crises, and spreads containment measures [2].

Complementarily, in crises, journalists react early, similar to other frontline professionals (i.e., police, firefighters, army, doctors) [3] and in these contexts, not only their journalistic experience is the key for looking forward and presenting information, but also their civic responsibility and the practice of the principles that govern the profession, along with skills to face adverse and potentially traumatic situations, in which they do not always have training [4,5,6]. In this context, experts agree that improving the training of cover crisis situations reduces the chances of journalist’s mental health being affected [7,8,9]; and, on the contrary, lack of knowledge on the part of employers and coworkers would be risk factors associated with mental health problems [10].

News production per se, contemplates a permanent exposure to trauma (i.e., seeing corpses), due to the usual pressure to satisfy the demand for critical information [11]. Journalists are frequently exposed to cover crisis situations; however, this could affect their mental health, since, in emergencies, there are usually: (1) few personnel with training to deal with them (i.e., safety and self-care measures), (2) scarce resources (i.e., protective equipment), (3) high emotional involvement, (4) excessive aversive stimulation, (5) contact with victims, (6) frustration for not being able to help, (7) role conflicts and ambiguity [12].

In addition to these factors, in crises, the working conditions of media companies could limit the action of journalists and contribute to increasing mental and/or physical conditions [13]. Among the labor factors that usually occur are: instability, low salaries, extended working hours, layoffs, and increase of functions, lack of resources for the development of coverage, lack of training on safety and biosafety standards, violence and aggressions they face in their productive routines [14,15]. In this regard the Ecuadorian NGO Fundamedios reported that in the last year at least 271 journalists in the country were dismissed, in addition to the closure of private media and the merger of 10 public companies including Public Media which includes the newspaper El Telégrafo, Radio Pública and Ecuador TV, printing presses and other media [16].

Despite the aforementioned risk factors, research suggests that journalists are resilient [5,17] and maintain adaptive coping styles, such as the satisfaction of a job well done, as well as considering that their work makes a difference in the results, which can be considered as protective factors [5]. However, although most journalists find ways to cope with crisis coverage [18,19], if their coping pathway is not adaptive, sooner or later it could take its toll on their mental health, especially if they have faced death-related situations [20]. In this sense, the International Center for Journalists (ICFJ) and the Tow Center for the Columbia University Digital Journalism launched an initiative in April 2020 to study the impacts of the new coronavirus crisis on journalism worldwide. The results are both alarming and disturbing. Based on the analysis of 1406 surveys completed during the first wave of the pandemic, numerous journalists who covered this serious human situation, at great risk to their person, had clear difficulties in dealing with it. In total, 70% of our respondents indicated that the most difficult aspect of her job was the psychological and emotional impact of dealing with the COVID-19 crisis [21].

### 1.1. Journalists as a Risk Group in Crisis Situations

The experts gathered at the Sixth Communication Forum, who were involved in the management of some worldwide crises (i.e., bird flu alert in Austria, SARS in Norway and in Ontario), suggest some lines of action for crisis communication, among them: (1) be proactive, (2) communicate in a simple, precise and transparent way, (3) be honest and avoid keeping secret information to reassure the public, (4) show respect and empathy with the public’s anxiety and (5) communicate accordingly [22,23]; however, these technical recommendations do not include self-care strategies, especially for mental health. The Pan American Health Organization (PAHO) launched in 2020 a guide for journalists on COVID-19 news coverage, this guide contains a series of tips for communicational work, but in the same way, self-care strategies and damage repair, mentions are limited [24]. This reveals how poorly visible the mental health risk that journalists are exposed to is and the stigma associated with mental health that some studies have reported, both in journalists and in their work contexts [25,26,27].

The low importance of mental health care for journalists exacerbates and intensifies frequent mental health problems such as: post-traumatic stress disorder [26], burnout [11], depression [26,27,28], anxiety, sleep disorders, social dysfunction [29,30] and somatizations (i.e., musculoskeletal problems, headaches, hypertension, gastrointestinal problems) [14,30,31]. Moreover, somatic complaints tend to be higher in journalists who have experienced critical situations [32]. In this context, this research seeks to show how journalism is a risk group in mental health and how mental disorders are associated with job change of journalists, especially in critical situations.

In this regard, since its creation in 1991, the Dart Center for Journalism and Trauma has collected a series of data about journalistic coverage of traumatic situations. Much of these data point to the experience of tragedy as a powerful incentive to abandon journalism, because “there is usually an emotional attachment between journalists and the events they cover”, but the harmful effects it has on the journalist are not always identified and addressed [33].

For crisis situations such as terrorist attacks, these coverages require unique journalistic standards [34]; even that in times of crisis certain journalistic roles should be different from those assumed when covering other topics of the daily news agenda [35]. Additionally, there is a difference between executing field work and desk work [36], therefore the media needs to properly identify the risk level of their staff to prevent psychological damage and deal with it in a timely manner.

The risk level of journalistic work exceeds their physical and mental conditions, and it can even cost them their lives. For example, in the last decade, at least 900 journalists have been killed in situations such as: armed clashes, drug trafficking, public protests, organized crime and others [37]. The Inter American Press Association (IAPA) reported that 24 journalists were killed and one was kidnapped during 2020 in the Americas [38]; and, in the last year more than 1000 journalists were killed while covering COVID-19 in 73 countries around the world according to the Press Emblem Campaign [39].

In the wake of the COVID-19 pandemic, the International Center for Journalists (ICFJ) and the Tow Center launched a global survey to study the effects of the Covid-19 pandemic on journalism. This study considers, in addition to the risks faced by journalists in obtaining information from the front line of the crisis, problems such as misinformation, disintegration of traditional business models and threats to media freedom and journalists [40].

The findings of the study reflect that the psychological and emotional impact is the most difficult aspect of journalistic work, making the current professional environment the most complex that most journalists have experienced in their careers, while highlighting the needs of professionals, which range from professional training, confronting current challenges such as disinformation and threats to freedom of expression, to improving working conditions [41].

A similar study, conducted by the Reuters Institute for the Study of Journalism and the University of Toronto, reflects that journalist covering the pandemic are more likely to develop anxiety and depression, and this is due to the coverage of painful situations involving human suffering, coupled with a drastic increase in work demands and lack of training for pandemic coverage [42].

This proves the fragility of the profession and its permanent risks, as a result of which many journalists are burned out, mainly due to lack of motivation (low personal accomplishment), because they no longer share the values of the company [43] and because they feel unprotected by their employers. Additionally, they suffer a high level of emotional exhaustion, due to the workload and with it the desire to change jobs [11].

### 1.2. Mental Health of Journalists

Being a journalist involves several risk factors regarding their mental health, among them: (1) the need to have updated data and news (especially in crisis situations), (2) high levels of competitiveness among them and (3) exposure to circumstances that endanger their integrity [10]. Additionally, the interactions that are established between journalists and victims, or friends and relatives of victims at the scene of events expose them to a situation of experiencing emotions for which they could not be prepared, which implies “relational transactions in the workplace” [44] (p. 400), ref. [7]. These are determinants for the development of burnout symptoms, such as those developed by health care workers (doctors, nurses, psychologists, etc.) [44,45].

However, it has also been found that some of the journalists report ineffective stress management in the workplace, in this scenarios they are exposed to a traumatic event (i.e., seeing a corpse) or threats with violence, which provoke physical and emotional effects related to their work performance [46,47], contributing this way to develop symptoms of post-traumatic stress disorder, depression and alcohol consumption [25], burnout [7,11,48] and compassion fatigue [7].

Part of the professional practice assumes that at some point journalists will assume risks that will affect their own health [49], although they do not always have access to services that allow them to meet their needs. In the case of mental health [50] they point out that there are limitations in access to therapy, and employers themselves do not consider it as a prescriptive element [31,51].

All these factors contribute to the possibility that a journalist may think about leaving their profession or at least seek a work environment in which them can be trained, protected, repaired and cared for in terms of mental health—this research attempts to determine which are the mental health factors that most influence the desire to change jobs. Additionally, this study aims to determine the prevalence of mental health problems and their association with perception of aptitude for covering emergencies and difficulty in seeing a corpse.

## 2. Materials and Methods

### 2.1. Method

This study was cross-sectional, prospective and observational, with a quantitative approach and correlational scope. Participation was confidential and completely anonymous. The study was approved by the Comité Expedito de Ética de Investigación en Seres Humanos (CEISH) of the Ministry of Public Health of the Republic of Ecuador (Code 009-2020; date of approval: 9 September 2020) and complies with the principles expressed in the Declaration of Helsinki [52]. Informed consent was obtained from all subjects involved in the study.

### 2.2. Participants

A total of 453 invitations to participate in the research process were sent out, and partial and complete responses were obtained from 196 (acceptance rate = 43.27%). The inclusion criteria were: journalists, social communicators or personnel who have worked or are currently working in the media, they freely agreed to participate in the study, and ensure being of legal age (over 18 years). A total of 196 journalists entered the analysis, who completed at least 75% of the psychological test, and in case of any instrument that was not completed, it was decided to replace the data by the average, in order to keep the 196 subjects without altering the results.

The participants in this study reported an average age of 33.65 years (SD = 8.16). Men and women were almost equally represented, 57.5% were single and resided in 22 provinces of Ecuador, the most represented being Pichincha, Loja and Guayas. In total, 9 out of 10 were of mixed race, more than the half were middle class or higher (65%) and 70.7% work outside the office (reporters, cameramen, photographers) (see Table 1).

### 2.3. Instruments

An ad hoc basic socio-demographic survey was designed to measure sociodemographic indicators such as: sex, age, province, marital status, ethnicity and socioeconomic level. Additionally, it was designed to collect factors related to the work environment: years of experience, job role (field work and office work), access to mental health services (social security, paid service, free service), perception of aptitude for covering emergencies (through the question, do I feel qualified to do hedging in risky contexts?), difficulty in seeing a corpse (through the question, is it hard for me to see a corpse?, and the desire to change job (through the question, do I feel like I want to change jobs or quit journalism?, the last three questions is responded with a four-point Likert scale (No agreement, little agreement, agreed, very much in agreement).

Three psychological instruments were applied:

1. General Health Questionnaire (GHQ-28) [53]: We used the version for the Spanish population of the GHQ-28 to evaluate the severity of a mental problem during the past few weeks using a four-point Likert scale. It has 28 items. This version is composed of four scales: somatic symptoms, anxiety and insomnia, social dysfunction (SDf) and major depression. Its concurrent validity suggests additional information on anxiety and depression, the cut-off point used is ≥5 (sensitivity 84–6%, specificity 82%), to determine whether it is a risk case;
2. Maslach Burnout Inventory (MBI-J). We used the version for journalists [54], based on Maslach’s burnout theory [44]. It is a 22-item self-administered scale that was used to measure burnout, and is based on three dimensions: emotional exhaustion (EE) (9 items), depersonalization (5 items), and personal accomplishment (PA) (8 items). Participants responded on a 7-point scale (0 = never and 6 = daily). A sample item was “My job has too many demands”. Internal consistency for this study was good, with a Cronbach’s α of α = 0.89 for EE, α = 0.60 for depersonalization and α = 0.71 for PA [53,54], The following cut-off points were used EE > 26; Depersonalization > 9 and PA < 14;
3. Brief scale to diagnose Post-Traumatic Stress Disorder [55,56], which consists of 12 items that are answered on a Likert scale from 0 (Not at all) to 3 (Very much). The cut-off points in relation to the level of emotional risk for post-traumatic stress are: Low presence of post-traumatic stress (0–3 points), Risk of post-traumatic stress (4–5 points) and post-traumatic stress (6–12 points). Internal consistency for this study was good, with a Cronbach’s α of α = 0.94. Item 12 refers to the possibility of suicidal risk, so it has its own interpretation, indicating that it should be reassessed (1 point), or intervention (2–3 points).

### 2.4. Procedure

Data were collected between 11 September 2020 and 15 January 2021. The research team sent via e-mail the invitation to participate in the study to media managers and journalists, with the link to access the online psychological test. The informed consent form was shown at the beginning, which could be revoked at any time. This test lasted approximately 30 min. The set of instruments consisted of one ad hoc survey and three psychological tests, which were organized, piloted and approved by the research team. No questions were mandatory. A brief summary of individual scores was provided for free after completing the test, to encourage honest responses and a higher participation rate. Invitations to participate in self-care workshops were also sent as part of the benefits. In no case was financial compensation offered.

### 2.5. Data Analysis

After cleaning the data matrix, descriptive analyses were performed using measures of central tendency and dispersion (continuous variables) and measures of frequency and percentage (categorical variables). To analyze the association between work factors and mental health variables (burnout, somatization, anxiety, social dysfunction, depression and post-traumatic stress). The association with continuous variables was performed through Student’s *t*-test and ANOVA for independent samples; and the association with categorical variables through Chi-square. Finally, a binary logistic regression was applied to determine the dichotomic mental health variables (0 = No case and 1 = case, for following values: SDF, depression, EE, depersonalization, PA, and SR) with predictive value of the desire to change jobs, for this the Likert scale was dichotomized as follows: 0 (no case) who responds with no agreement or little agreement to the question: I feel like I want to change jobs or quit journalism? and 1 (case) who responds with agreed or very much in agreement. The significance value was taken as *p* < 0.05. SPSS-23 IMB^®^ software was used.

## 3. Results

According to the cut-off points of each instrument (see methodology), it has been determined that the main mental health problems are low personal accomplishment (PA) and emotional exhaustion (EE), present in one out of six journalists. Regarding the risk of other mental health problems, 11.2% presented symptomatology is related to Post-Traumatic Stress Disorder, 7.7% to risk of Anxiety and Insomnia and 7% to Suicidal Risk. The remaining mental health problems are present with a prevalence of less than 6.1% (somatization, depression, social dysfunction) only one journalist had burnout (see Table 2).

In total, 42.1% of journalists could only access mental health services if they were free of charge. A total of 71.9% of those evaluated perceive themselves as having an adequate aptitude for journalistic coverage in risk situations, and 28% report having difficulties when seeing corpse. Finally, 17.3% of journalists consider that they have the desire to change jobs (See Table 3). Additionally, a chi square was applied to identify the association between those with mental health problems, sex (male and female) and the type of role (field work and office work) of the journalist, and none was found to be significant.

When running the correlational analysis it was found that: (1) age is inversely related to anxiety and insomnia (R = −0.176, *p* < 0.05) and post-traumatic stress disorder (R = −0.152, *p* < 0.05); (2) years of work experience did not show significant correlations; (3) perceived aptitude to cover emergencies is inversely related to SDf (Rho = −0.173, *p* < 0.05) (4) years of work experience showed no significant correlations; (5) perceived aptitude to cover emergencies is inversely related to SDf (Rho = −0.173, *p* < 0.05), EE (Rho = −0.264, *p* < 0.001), and directly related to PA (Rho = −0.246, *p* < 0.001); (4) difficulty seeing corpse correlates directly with anxiety and insomnia symptoms (Rho = 0.156, *p* < 0.05) and SR (Rho = 0.247, *p* < 0.001); (5) the desire to change jobs has a direct relationship with SDf (Rho = 0.166, *p* < 0.05), EE (Rho= 0.326, *p* < 0.001), and inversely related to PA (Rho = −0.253, *p* < 0.001), post-traumatic stress disorder symptoms (Rho = 0.225, *p* < 0.001) and SR (Rho = 0.198, *p* < 0.05) (see Table 4).

When running the Student’s *t*-test for mean differences between journalists who are sure of continuing in their jobs and those who are insecure, it was found that: (1) there are significant differences in SDf (T = −2.82, *p* = 0.005), with dysfunction being higher in journalists who want desire to change jobs; (2) there are significant differences in depression symptomatology (T = −2.99, *p* = 0.003), with depression being higher in journalists who desire to change jobs; (3) there are significant differences in EE (T = −4.50, *p* < 0.001), with greater emotional exhaustion among journalists who desire to change jobs; (4) there are significant differences in depersonalization (T = −2.56, *p* = 0.011), with greater depersonalization among journalists who desire to change jobs; (5) there are significant differences in PA (T = 4.60, *p* < 0.001), and a low personal accomplishment in journalists who desire to change jobs; (6) there are significant differences in post-traumatic stress disorder (T = −3.051, *p* = 0.003), with a greater Post-Traumatic Stress Disorder symptomatology in journalists who desire to change jobs. When running a Chi-square test, it was determined that there is a significant association between suicidal risk and the desire to change jobs (χ = 6.795, *p = 0*.009), with a higher proportion of journalists with suicidal risk in the group who desire to change jobs (see Table 5).

In order to obtain the best adjustment and most parsimonious model, we simultaneously analyzed the categorical mental health values (0 = no case and 1 = case, for following values: SDF, depression, EE, depersonalization, PA, and SR), it was found that journalists with higher EE (adjusted OR = 3.268, 95% CI [1.021, 10.453]) and low PA (adjusted OR = 0.107; 95% CI [0.013, 0.855]) have a greater desire to change jobs or quit being a journalist (See Table 6).

## 4. Discussion

In recent decades, there has been a need to explore aspects related to mental health in the workplace, and probably one of the least explored is journalism, which is why some mental health problems are considered, in order to identify the degree of vulnerability in the profession, especially about the coverage of critical situations.

Some aspects have been identified that could have an impact on the mental health of Ecuadorian journalists, about one-third of those evaluated reported that they have problems in seeing a corpse or have a low aptitude in carrying out journalistic coverage in risky situations. This is congruent with the low importance or even stigma attached to mental health issues in journalists and their work contexts [24,25,26,27]. In addition, it was found that low perceived aptitude to cover emergencies would be associated with greater emotional exhaustion, low personal accomplishment and social dysfunction. While the greater the difficulty in seeing corpse, the higher the scores in suicidal risk, anxiety and insomnia, this would be consistent with the low importance given to mental health issues in university education [4], which would increase the risk of mental health problems in their graduates. Furthermore, in addition to their vulnerability, there is difficulty in accessing mental health services: it is reported that 4 out of 10 Ecuadorian journalists could only access these services free of charge, and if the lack of attention to mental health is maintained, it probably leads to an increase in cases of suicide risk (more than 7% found).

The data report that the prevention of Post-Traumatic Stress Disorder, Anxiety and Insomnia should be a priority in younger journalists, which is partially similar reported in previous studies [32], which argues that more experienced journalists are the risk group for post-traumatic stress. This paradoxical result could be related to the overexposure of new generations of journalists to information about crisis situations. Social support is a protective factor in the mental health of journalists [51,57]. This implies the need to improve the social sphere of support not just in work contexts [31,51], but also outside them [31,57].

The data collection took place in a time of health crisis in Ecuador (COVID-19 pandemic). In normal situations it has already been reported that journalism is a profession with a high prevalence of mental health problems [26], and that an increase would be expected in crisis situations. In this sample, high prevalence of symptoms associated with burnout, such as low personal accomplishment (17.3%) and emotional exhaustion (16.3%) are reported, which are very similar to those reported in health workers in Ecuador [45]. In non-crisis situations, this could be associated with deficiencies in their training to cover emergencies and that one out of eight perceives low social support—this factor has demonstrated its ability to influence emotional exhaustion in health workers in Ecuador [55]. Finally, Post-Traumatic Stress Disorder also occurs with a prevalence of 11.2%, which is much higher than that reported (7.2%) in the meta-analysis of [25]. The symptoms of PTSD are greater in journalists who report increased difficulty in seeing a corpse, which would ratify what was reported by [20]. The rest of the mental health problems are present with a prevalence of less than 6.1% (somatization, depression, social dysfunction), consistent with that reported by other authors [31] who used the GHQ-28 in journalists.

Seventeen percent of journalists desire to change jobs, and this is related to greater social dysfunction, depression, emotional exhaustion, depersonalization, low personal accomplishment, post-traumatic stress symptomatology and suicidal risk in the group of journalists who desire to change jobs. However, only low personal accomplishment and emotional exhaustion showed the capacity to predict change jobs (see Table 4, Table 5 and Table 6). These results reaffirm the need to prioritize the prevention or repairment of the mental health problems in journalists, not only for individual well-being, but also for the working environment of media companies and for the optimization of training resources, employee retention and job security [7]. In addition, the employment outlook for the country’s journalists is uncertain, given the growing layoffs of journalists and the closure of media outlets, factors that contribute to the desire to change jobs in relation to the feeling of low personal accomplishment (17.3%).

Two relevant intervention topics are Post-Traumatic Stress Disorder and two of its comorbidities (depression and suicidal risk) [25], and prevention and timely attention on burnout syndrome, consistent with that reported in previous research [10]. This is especially important to detect low personal accomplishment and emotional exhaustion as suggested in the studies of [46], and to comply with occupational safety policies. In addition, this study used an adaptation of the MBI, for journalists that could facilitate early detection, although there are indications that the MBI-GS also has good psychometric indicators in journalists [58].

One of the limitations of this study is that it is a convenience sample, and that its recruitment was influenced by the voluntary nature of answering the psychological test and access to the Internet. In addition, the use of two instruments adapted to the Ecuadorian population [54,56] would make comparison with studies from other latitudes difficult. Finally, another limitation is not having information on the prior mental health of journalists.

In the future, the sample of journalists can be expanded to look for more specific protective and risk factors for emotional exhaustion and low personal accomplishment, among them, the family and work social support of journalists and the organizational climate. Additionally, in this manner, longitudinal studies are needed in young journalists who work as freelancers or without an employment relationship, as well as in journalists who cover red chronicles or in crisis situations.

This study opens the gate to start evaluating university, post-university and work training processes, focusing on the development of self-care competencies in mental health for journalists.

The information resulting from this study may be useful in the design of public policies aimed at improving the working conditions of journalists in the country and increasing good occupational health practices in the media, in relation to mental health issues, early detection of symptoms, continuous training in the coverage of risk situations, among others.

## 5. Conclusions

The Ecuadorian journalists have great difficulty accessing mental health services, and would only be able if it were free or paid for social security.

About one-third of Ecuadorian journalists have problems with seeing a corpse or a low aptitude to cover journalistic coverage in risky situations. The low perception of aptitude to cover emergencies would be associated with increased emotional exhaustion, low personal accomplishment and social dysfunction. Further, the bigger the difficulty in seeing corpse, the higher the scores for suicidal risk, anxiety and insomnia.

The data report that prevention of Post-Traumatic Stress Disorder, anxiety and insomnia should be a priority in younger journalists.

Ecuadorian journalists report a high prevalence of burnout symptoms such as low personal accomplishment and emotional exhaustion.

Post-Traumatic Stress Disorder symptoms are higher in journalists who report greater difficulty in seeing a corpse. In addition, two comorbidities with Post-Traumatic Stress Disorder are also of interest, such as suicidal risk and depressive symptomatology.

Seventeen percent of journalists desire to change jobs, and this is more related to: social dysfunction, depression, emotional exhaustion, depersonalization, low personal accomplishment, post-traumatic stress disorder symptomatology and suicidal risk. However, only professional frustration and emotional exhaustion showed the ability to predict the desire to change jobs.

## Figures and Tables

**Table 1:** Sociodemographic data.

Sociodemographic Variables	*n* (196)	M (Range)	SD
Age		33.65 (21–62)	8.16
		**f**	**%**
Sex *	Male	93	48.4
	Female	99	51.6
Provinces **	Pichincha	45	23.3
	Loja	36	18.7
	Guayas	22	11.4
	El Oro	14	7.3
	Rest of Country	79	39.3
Marital status **	Single	111	57.5
	Married/free union	67	34.7
	Divorced/separated	14	7.3
	Widower	1	0.5
Ethnicity **	Mixed	178	92.2
	Minorities	15	7.8
Socioeconomic level **	Low	14	7.3
	Medium Low	52	26.9
	Medium	113	58.5
	Medium high	12	6.2
	High	2	1.0
Role ***	Field work	116	70.7
	Office work	48	29.3

Note: * *n* = 192; ** *n* = 193; *** *n* = 164.

**Table 2:** Prevalence of mental health.

Mental Health Variables	Risk		No Risk			
	f	%	F		%	
Somatization	12	6.1	184		93.9	
Anxiety/insomnia	15	7.7	181		92.3	
Social dysfunction	4	2.0	192		98.0	
Depression	11	5.6	185		94.4	
Suicidal risk *	8	7.0	107		93.0	
Emotional exhaustion	32	16.3	164		83.7	
Depersonalization	21	10.7	175		89.3	
Low personal accomplishment	34	17.3	162		82.7	
Burnout	1	0.5	195		99.5	
	**Case**		**Risk**		**No risk**	
	**f**	**%**	**f**	**%**	**f**	**%**
Post-Traumatic Stress Disorder	22	11.2	120	61.2	54	27.6

Note: * *n* = 115.

**Table 3:** Factors related to the journalist’s work.

Labor Variables	*n* (196)	*f*	%
Access to mental health services.	Social Security.	51	31.1
	Paid service.	44	26.8
	Free service.	69	42.1
Perception of aptitude for covering emergencies.	No agreement.	14	7.1
	Little agreement.	47	24.0
	Agreed.	115	56.6
	Very much in agreement.	30	15.3
Perception of difficulty in seeing a corpse.	No agreement.	41	20.9
	Little agreement.	100	51.0
	Agreed.	33	16.8
	Very much in agreement.	22	11.2
Desire to change jobs.	No agreement.	109	55.6
	Little agreement.	53	27.0
	Agreed.	22	11.2
	Very much in agreement.	12	6.1

**Table 4:** Correlations between mental health and labor variables.

Mental Health Variables	Age	Experience	Aptitude to Cover Emergencies	Difficulty to Seeing Corpse	Desire to Change Jobs
	R (Pearson)	R (Pearson)	Rho (Sperman)	Rho (Sperman)	Rho (Sperman)
Somatization	−0.04	0.113	−0.110	0.113	0.108
Anxiety/insomnia	−0.176 *	−0.109	−0.055	0.156 *	0.145
Social dysfunction	−0.089	−0.038	−0.173 *	0.140	0.166 *
Depression	−0.117	−0.112	−0.012	0.076	0.088
Emotional exhaustion	−0.140	−0.100	−0.264 **	0.241	0.326 **
Depersonalization	−0.041	−0.015	0.020	−0.012	0.110
Personal accomplishment	0.062	0.074	0.246 **	−0.086	−0.253 **
Post-Traumatic Stress Disorder	−0.152 *	−0.114	−0.080	0.187*	0.225 **
	Rho (Sperman)	Rho (Sperman)	Rho (Sperman)	Rho (Sperman)	Rho (Sperman)
Suicide risk	−0.105	−0.097	−0.016	0.247 **	0.198 *

Note: * *p* < 0.05; ** *p* < 0.01.

**Table 5:** Mean differences according to desire change job and mental health.

Mental Health Variables	Has No Desire to Change Jobs		Has the Desire to Change Jobs		*T*	*p*
	*Media*	*SD*	*Media*	*SD*		
Somatization	2.52	1.45	2.86	2.03	−1.13	0.258
Anxiety/insomnia	1.99	1.90	2.66	1.74	−1.88	0.062
Social dysfunction	1.42	1.43	2.25	1.89	−2.82	0.005
Depression	1.03	1.51	2.04	2.5	−2.99	0.003
Emotional exhaustion	19.56	10.23	28.92	12.76	−4.50	0.000
Depersonalization	5.10	3.89	7.29	6.12	−2.56	0.011
Personal accomplishment	39.53	5.01	34.29	8.57	4.60	0.000
Post-Traumatic Stress Disorder	10.39	6.47	14.12	5.83	−3.051	0.003
	**f**	**%**	**f**	**%**	**χ**	***p***
Suicide risk	3	3.4	5	17.9	6.795	0.009
No Suicide Risk	84	96.6	23	82.1		

**Table 6:** Mental health risk factors for job change.

Mental Health Variables	Adjusted OR	95% CI	
		Lower Limit	Upper Limit
Social dysfunction	0.121	0.006	2.400
Depression	3.775	0.372	37.947
Emotional exhaustion *	3.268	1.021	10.453
Depersonalization	2.131	0.616	7.371
Low personal accomplishment *	0.107	0.013	0.855
Suicide risk	2.507	0.306	20.56

Note: * *p* < 0.05.

## Data Availability

The data for this study were stored and encrypted through UTPL’s TrueTest software, were collected through the following link: https://truetest.utpl.edu.ec/project-published/Tm2PaTMz9ysqv2H1YwcsJ8 (accessed on 11 September 2020) and the raw data have been stored in the following link: https://truetest.utpl.edu.ec/results?filter=Published (accessed on 15 January 2021) and have been cleaned and processed in the SPSS 23 software whose data are available to the principal investigator of the project and who is in charge of their custody.
